# Comparison of nutritional intake in US adolescent swimmers and non-athletes

**DOI:** 10.4236/health.2012.410133

**Published:** 2012-10

**Authors:** Andy C. Collins, Kenneth D. Ward, Bridget Mirza, Deborah L. Slawson, Barbara S. McClanahan, Christopher Vukadinovich

**Affiliations:** 1 Division of Social and Behavioral Sciences, School of Public Health, The University of Memphis, Memphis, USA; 2 Memphis, USA; 3 College of Public Health, East Tennessee State University, Johnson City, USA; 4 Department of Health and Sport Sciences, The University of Memphis, Memphis, USA; 5 Department of Epidemiology & Cancer Control, St Jude Children’s Research Hospital, Memphis, USA

**Keywords:** Nutrition, Dietary Intake, Swimming, Bone Health

## Abstract

Swimming is a very popular sport among adolescents in the US. Little is known about the diet of competitive adolescent swimmers in the US but data from other countries indicate several inadequacies, including excessive intake of fat and lower than recommended intake of carbohydrate and several micronutrients that may affect athletic performance and bone accrual. We assessed usual diet, using a food frequency questionnaire and calcium checklist, among 191 adolescent males and females [91 swimmers (mean 13.7, s = 2.5 years) and 100 non-athletes (mean 14.4, s = 2.8 years)]. For both males and females, swimmers and non-athletes generally had similar average intakes of macro- and micro-nutrients, including higher than recommended amounts of total fat (36%) and saturated fat (12%), and inadequate amounts of calcium, vitamin D, and daily servings of fruits, vegetables, grains, and dairy products. This first study of nutritional intake among adolescent swimmers in the US suggests that dietary habits of adolescents who swim competitively may jeopardize optimal athletic performance and place them at risk for future chronic diseases, including osteoporosis.

## 1. INTRODUCTION

Swimming is a popular sport among adolescents in many countries. In the US, over two hundred fifty thousand youth participate each year in club, school, and summer league teams [1]. Competitive swimming has high-energy demands (25% to 100% higher compared to non-swimmers) that must be matched by a balanced diet to optimize physical performance [2-4]. Given that adolescence is a time of increased nutritional demands to meet growth, as well as a time when nutritional adequacy often dissipates [5], maintaining a balanced dietary intake may be especially problematic for adolescent swimmers.

Several studies of collegiate and college-age elite swimmers indicate that their diets are typically high in fat (over 35%) and cholesterol (over 300 mg), low in carbohydrate (below 50%), and often deficient in several micronutrients, including calcium, zinc, and iron [6-8] relative to American Dietetic Association, Dietitians of Canada, and American College of Sports Medicine recommendations for adult athletes [4]. Standard dietary guidelines are not available for adolescent swimmers or adolescent athletes in general. However, available recommendations are that this population should adhere to national nutritional recommendations for adolescents: that at least 50% of caloric intake comes from carbohydrates, less than 30% from fat, and 10% - 15% from protein [9]. Energy and micronutrient requirements of adolescent athletes are higher than non-athletes. In particular, carbohydrate needs are greater in athletes due to greater energy expenditure [4]. Further, because bone accrual is accelerated during puberty, adequate intake of calcium, iron, and zinc is important [4].

Research on the nutritional status of adolescent swimmers is scarce, but available data generally parallel results from college-age swimmers. Among 20 (9 boys, 11 girls) competitive swimmers in Australia (mean age 13 years), girls’ intake of carbohydrate, calcium, and iron were below recommended levels [3]. Similarly, among 35 (20 boys, 15 girls) 15 - 18 year old Greek elite swimmers, fat intake was higher and carbohydrate intake lower than recommended levels, although intake of calcium, iron, and zinc were adequate [10]. Thirty six (22 boys, 14 girls) semi-professional adolescent swimmers in Spain had lower than recommended levels of total energy intake and several micronutrients, including vitamin D, and girls had inadequate calcium and iron intake [11]. In the only published study from the US, 43 (22 boys, 21 girls) adolescent elite swimmers participating in a national developmental training camp were found to have diets comparable to the general population of US adolescents, which included higher than recommended percent of calories from fat and lower than recommended carbohydrate. Calcium intake was deficient in 52% of girls and 14% of boys [2]. Limitations of this study are that diet was assessed only during training camp which may be different from normal and included only national elite swimmers, whose diet may differ from competitive swimmers who do not participate at this level.

Thus, the limited available data suggest that macro- and micronutrient intake of adolescent competitive swimmers may be similar to that of the general population of adolescents and inadequate to ensure adequate growth, bone development, and athletic performance. The aim of this study is to compare the nutritional adequacy of adolescent competitive swimmers and non-athletes in Memphis, Tennessee.

## 2. METHODS

### 2.1. Participants

Participants were enrolled in a prospective observational study of lifestyle factors affecting bone development of child and adolescent competitive swimmers and non-athletes. A total of 131 swimmers and 200 non-swimmers, 8 - 18 years of age were recruited between 2000 and 2002 from the Memphis area. For the present study, baseline data were used from non-Hispanic white participants who were in puberty. Analyses were restricted to non-Hispanic whites due to low enrollment of ethnic minority swimmers. Of the 331 participants, 71 were excluded from the present study because they were pre-pubertal (35 swimmers, and 36 non-athletes), and an additional 69 ethnic minorities (5 swimmers, and 64 non-athletes) were excluded, resulting in a sample of 191 (91 swimmers, and 100 non-swimmers).

Competitive swimmers were defined as those who were members of a competitive team (USA Swimming, school teams, or club teams) for at least the past year and planned to continue to compete on a swim team for the next two years. They were recruited through the coaching staffs of teams and at local swim meets. Non-athletes were defined as those who did not engage in at least 20 minutes of physical activity three or more days per week and had not participated in organized athletic activities for at least one year. Non-athletes were recruited through flyer distributions at grocery stores and libraries, newspaper articles, and telephone “on hold” advertisements at a Memphis-area university.

### 2.2. Procedure

Parents of potential participants were screened over the phone for eligibility and invited to a university laboratory with their child where study goals and requirements were explained in detail and eligibility verified. Parents of children 17 years of age and younger provided written consent, and children provided written assent. Eighteen-year-old participants provided written consent. Parents and participants then completed several self-report measures. When paperwork was completed, a research assistant collected height and weight of each subject. Participants then privately completed a self-assessment of pubertal development. All consent/assent documents and procedures were approved by the Institutional Review Board at The University of Memphis.

### 2.3. Measures

#### Sociodemographics

Age, gender, and race were collected through a demographic survey.

#### Height and Weight

Height and weight of each participant were collected by a trained research assistant following standardized procedures. Height was measured to the nearest quarter-inch with a wall-mounted stadiometer. Weight was measured without shoes and outer clothing on a calibrated beam-balance scale. Body Mass Index (BMI) was calculated by dividing a participant’s body weight (kg) by the square of his or her height (m).

#### Pubertal Status

Pubertal status was self-assessed using standardized drawings and descriptions based on the Tanner stages of pubertal maturation [12,13]. Male participants rated their stage of pubertal development by choosing one of five illustrations that most closely resembled their pubic hair and genitalia. Female participants rated their stage of pubertal development by choosing from two sets of illustrations, one for breast development and one for pubic hair development. Self-reported pubertal status by 9 - 17 year olds using Tanner staging agrees very highly with physician ratings via physical examination, with kappa coefficients for female breast stage, female pubic hair stage, and male combined pubic hair and genital stage ratings of 0.81, 0.91, and 0.88, respectively [14]. For analyses, participants were subdivided into three pubertal groups: prepuberty (Tanner stage 1 for breasts or genitalia and pubic hair), early puberty (Tanner stage 2 and 3 for breasts or genitalia), and late puberty (Tanner stage 4 and 5 for breasts or genitalia) [15]. Only participants in early or late puberty were retained.

#### Physical Activity

Swimmers reported, with assistance from parents, the number of weeks during the past year of participating on a swim team, and the average number of hours per week, including both practices and meets. Swimmers and non-athletes completed, with assistance from parents, the Seven Day Physical Activity Recall (PAR) to estimate participants’ time spent in physical activity in the past week [16]. The PAR is designed to assess a variety of activities of moderate or greater intensity. Hours spent each day in moderate, intense, and very intense activities were calculated, from which total kilo-calories/day were estimated. The PAR has adequate test-retest reliability and validity compared to heart rate monitoring with adolescents [17].

#### Dietary Intake

Usual dietary consumption during the previous 12 months was assessed with the Block 98 semi-quantitative Food Frequency Questionnaire (FFQ). The Block 98 is a revision of the original Block FFQ developed at the National Cancer Institute [18]. Block questionnaires (paper format version) purchased from NutritionQuest, were administered to study participants and their parents (NutritionQuest, Berkley, California). After completion, forms were sent to NutritionQuest for dietary intake assessment.

The Block 98 includes 109 questions regarding typical food intake and supplement use over the past year. Food items are used that comprise more than 90% of the population intake for energy and nutrients. Average daily nutrient intake is obtained from a FFQ nutritional analysis based on reported frequency of consumption and portion sizes for each individual.

In a study of 25 - 74 year old adults [19], the Block 98 had good test-retest reliability, with a median correlation of 0.75, ranging from 0.57 - 0.90 for macronutrients and 0.65 - 0.88 for micronutrients. Validity, compared to dietary recall, was moderate to high. Using deattenuated correlation coefficients (*i.e*., corrected for attenuation due to random error in within-person variability, to allow a reasonable estimate of true correlation), overall median correlation across nutrients was 0.59. For the nutrients described in the current study, the lowest reported validity coefficient was 0.11 for cholesterol [19]; correlations for other nutrients ranged from 0.41 (for protein and fat expressed in g/day), to 0.73 for carbohydrate (expressed as % of total energy). Mean intakes from the FFQ and diet records were similar for total energy and most micronutrients, including calcium, iron, zinc, and vitamin D. The FFQ provided somewhat lower estimated means than diet records for carbohydrate, protein, saturated fat and cholesterol, and higher estimates for total fat. In another study of adult women [20] intake estimates for total energy and calcium were similar for the Block 98 and 5 days of food records. The Block 98 has not been validated in adolescents. For very young children (4 - 9 years of age) in which parents completed the Block 98 with assistance from the child, the instrument was moderately correlated with 3 days of diet records for total energy intake and macronutrients (r = 0.40 to 0.55) but overestimated intakes [21]. To increase accuracy in the current study, detailed instructions were given, portion size photographs were provided, participants were assisted by parents, and responses were checked with parents and participants before leaving the laboratory. From the FFQ, we derived estimates of usual daily intake of total energy (kcals/day), total fat, saturated fat, protein and carbohydrate (expressed as both grams/day and grams/kg body weight/day); fiber and cholesterol; number of servings of vegetables, grains, fruits, and dairy; and iron, zinc, vitamin D, and calcium.

We also assessed calcium intake over the past seven days using a modified version of the Rapid Assessment Method (RAM), a dietary checklist [22]. The original RAM lists common sources of calcium in the American diet and was developed using information from the National Health and Nutrition Examination Survey (NHANES) III. The RAM was modified to include calcium-fortified foods (e.g., orange juice and bread) that were introduced to the market after the development of the original RAM, the actual (rather than usual) number of servings of foods, and foods frequently eaten among athletes (such as bagels and baked potatoes). Serving sizes were illustrated with two-dimensional line drawings. The number of servings per week of each food was multiplied by the corresponding calcium value for that food, values were added together and divided by seven to obtain daily calcium intake over the past week. Adequate test-retest reliability (intraclass correlation = 0.54) and validity (assessed as agreement with 6 days of diet records; intraclass correlation = 0.41) were found for the modified RAM among a sample of college athletes [23]. As with the Block FFQ, a parent assisted the participant in completing the RAM and responses were verified before they left the laboratory.

#### Diet Adequacy

Adequacy of intake of micronutrients (iron, zinc, vitamin D, and calcium) was judged according to Dietary Reference Intakes (DRI) [24,25] using a classification scheme [8]. For each micronutrient, intakes below the EAR were considered “inadequate”; intakes at the EAR but below the RDA were considered “uncertain”, and intakes at or above the RDA were considered “adequate”.

### 2.4. Approach to Data Analysis

Histograms and descriptive statistics were generated on all variables to assess normality. T-tests were conducted to compare mean intake of nutrients in swimmers and non-athletes, stratified by gender.

## 3. RESULTS

### 3.1. Descriptives

The sample (n = 191) included 30 male and 61 female swimmers, and 39 male and 61 female non-athletes. Swimmers had engaged in competitive swimming for a mean of 4.3 years (s = 2.4 years, range = 1 - 12 years). Including both practice and meets, they swam an average of 37.6 weeks per year (s = 16.7, range = 4 - 52 weeks) and 8.6 hours/week across the whole year (s = 5.2 hours/week, range = 1.5 - 24 hours/week) (Table 1). Compared to non-athletes, swimmers were about six months younger on average (*p* = 0.058), more likely to be in early puberty, had lower body weight and BMI, and expended more energy per kg body weight (Table 1).

### 3.2. Macronutrients

For both males and females, neither total energy intake, nor total energy intake adjusted for body mass, differed significantly for swimmers and non-athletes (Table 2). Likewise, macronutrients (fat, saturated fat, carbohydrate, and protein) expressed as percent of total energy, did not differ significantly between swimmers and non-athletes, for either males or females (Table 2).

For all four swim status/gender groups, percent of calories from carbohydrate was slightly above 50%, consistent with the 45% - 65% recommended range for children. Total fat averaged approximately 36% in the four groups which was above the 25% - 35% recommended range, and protein averaged 13% - 14% which was within the 10% - 30% recommended range [24].

Macronutrient intake also was expressed as grams per kg body mass to adjust for body mass differences between swimmers and non-athletes. Total fat and saturated fat did not differ between swimmers and non-athletes for either males or females. Protein intake was higher among swimmers than non-athletes for both males and females, and carbohydrate intake was higher for male swimmers than non-athletes (Table 2). Protein intake was consistent with recommended intake range of 1.2 - 1.7 g/kg for adult endurance and strength athletes [4]. Carbohydrate intake for male swimmers (6.4 g/kg) was within the recommended range for adult athletes of 6 - 10 g/kg, but intake for female swimmers (5.5 g/kg) was below this recommended range. Fat intake of 35.9% of total energy, observed for both male and female swimmers, was higher than the recommended range for adult athletes of 20% - 35% [4].

### 3.3 Micronutrients

Micronutrient intake by swim status and gender is shown in Table 2. Among males, mean iron intake was greater in swimmers (20 mg/day) than non-swimmers (16 mg/day), although intake by both groups exceeded the 5.9 mg/day EAR for 9 - 13 year old males and 7.7 mg/day EAR for 14 - 18 years old males [24]. Iron intake was judged to be adequate for 93% of male swimmers and 62% of male non-athletes (Table 3). For females, mean iron intake did not differ significantly between swimmers and non-athletes and was above EAR of 5.7 mg/day for 9 - 13 year old females and 7.9 mg/day for 14 - 18 year old females. Iron intake was adequate for 62% of female swimmers and 51% of female non-athletes.

Mean zinc intake did not differ between swimmers and non-athletes for either males or females, and was consistent with EARs (7.0 mg/day for 9 - 13 year old males and females, 7.3 mg/day for 14 - 18 year old females, and 8.5 mg/day for 14 - 18 year old females) [24]. A majority of swimmers have adequate zinc intake (77% of males and 60% of females), compared to 33% and 43% of male and female non-athletes, respectively.

Past year and past week mean calcium intakes were very similar. Male swimmers and non-athletes did not differ significantly on either past year or past week intake, whereas female non-athletes had significantly lower intake than swimmers (Table 2). Mean levels were close to the calcium EAR of 1100 mg/day for all groups except female non-athletes, but only a small proportion (10% - 30%) of subjects in any of the four groups were judged to definitely have adequate intake, which was defined in this study as at or above the RDA of 1300 mg/day [25].

Mean daily intake of vitamin D did not differ by swim status and averaged approximately 6.0 μg and 5.0 μg for males and females, respectively, much lower than the 10 μg EAR for vitamin D [25]. Across the four groups, only 0% - 7% of subjects have adequate intake of vitamin D. Cholesterol intake averaged 211 mg/day and did not differ by swim status for either males or females. Fiber intake also did not differ between swimmers and non-swimmmers for either gender, but the intake of all four groups (approximately 16 g/day) was above the adequate intake (AI) level of 14 g/day for 9 - 18 year old females and males [24].

### 3.4. Food Group Servings per Day

Swimmers and non-athletes did not differ in number of servings of vegetables or grain, among both males and females (Table 2). Female swimmers consumed a greater number servings/day of fruits and dairy than non-athletes. Male swimmers and non-athletes did not differ in number of servings per day of vegetables, grains, fruits, or dairy. All four groups were below USDA recommended servings/day for vegetables, grains, fruits, and dairy [26].

## 4. DISCUSSION

This study indicates that diets of adolescent swimmers are generally similar to those of non-athletes. Both swimmers and non-athletes consumed higher than recommended amounts of total fat and saturated fat, and inadequate amounts of calcium, vitamin D, and daily servings of fruit, vegetables, grains, and dairy. Our finding that adolescent swimmers and non-athletes have broadly similar diets is consistent with other studies of adolescent swimmers [2,10] and college-age swimmers [8].

Given the increased energy and nutrient needs of adolescent athletes compared to the general population, these findings are concerning. There are no authoritative recommendations for macronutrient intake by adolescent athletes as there are for adult athletes [4]. Research on muscle glycogen utilization in adolescent athletes, however, indicates that carbohydrate is an important fuel to optimize athletic performance and recovery [9], suggesting that a higher-carbohydrate diet may be required by adolescent athletes than is recommended for the general population [27]. Carbohydrate intake of athletes in most sports is suggested to comprise 50% - 55% of total caloric intake [28]. Also problematic is our finding of excessive fat intake and inadequate fruit, vegetables, and whole grains, which increase the risk of obesity, numerous chronic diseases including heart disease, and premature death [29].

Although numerous micronutrients may affect athletic performance, we focused on calcium, vitamin D, zinc, and iron, since these play important roles in bone accrual and adolescent swimmers may be at risk of suboptimal bone development. Approximately 26% of bone mineral is accrued during adolescence [30]. Several studies have found that young competitive swimmers have lower bone mineral density (BMD) compared to athletes engaged in impact loading sports [31,32]. Other studies have reported that swimmers, compared to non-athletes, have similar or lower BMD [32,33]. These studies suggest that swimming provides insufficient skeletal loading, compared to resistance or weight-bearing activities, to promote BMD growth, and may actually impede the achievement of maximal peak bone density. Because calcium is an important modifiable determinant of BMD [34], ensuring optimal calcium intake in adolescents is important. In the current study, calcium intake was higher in swimmers than non-athletes, but less than one third of both male and female swimmers had adequate calcium intake. Estimates of adequate intake are based on requirements for the general population of this age group. Optimal calcium intake levels for adolescent athletes are not known, but may be higher than that of the general population. Based on the bone density studies reviewed above, however, it appears that current average intakes are inadequate to optimize bone accrual in swimmers. In this regard, the low number of servings of dairy products observed in this study is concerning, since dairy products are a major source of calcium in the American diet [27].

Virtually no subjects, either swimmers or non-athletes, had adequate intake of vitamin D, which plays several important roles in bone accrual, including promoting calcium absorption [35]. More encouraging was our finding that iron and zinc intake appeared adequate in a majority of both swimmers and non-athletes, both male and female. Zinc and iron play important roles in promoting athletic performance [36]. In addition, zinc stimulates bone mineralization [37]. Several enzymes involved in bone formation require iron to function properly, and iron deficiency is associated with bone density deficits [38].

This study makes a contribution to the literature by evaluating dietary adequacy among adolescent competitive swimmers in the US, who have received little research attention despite evidence of nutritional deficiencies, particularly related to bone accrual. Several limitations should be noted, however. First, data were collected from 2000-2002 and it is possible that dietary intake patterns of adolescents have changed since then. However, we are not aware of data or training recommendations indicating that changes have occurred either in the diets of adolescent swimmers or in total energy or macro-nutrient intake among the general population of US adolescents between 2000 and 2008, based on NHANES data [39]. NHANES data do reveal, however, an increase in calcium intake of approximately 200 mg/day for adolescent males (from 1003 mg/day in 2000 to 1205 mg/day in 2008) and 116 mg/day for among adolescent females (from 750 mg/day in 2000 to 866 mg/day in 2008) [39]. Thus, to the extent that dietary patterns in adolescent swimmers are similar to the general population, calcium intake may have increased in recent years, but is still below recommended levels.

Second, usual diet was assessed using an FFQ, which has limitations in terms of reliability and validity, especially among children [40]. The FFQ used in this study, however, has been widely used and demonstrates reasonable agreement with dietary records. Several steps were taken to maximize accurate reporting, including providing careful instructions and portion size photographs, having parents assist participants in completing the survey, and checking all responses with the parent and participant. Additional limitations are that convenience sampling was used to recruit participants, who resided in a single region in the US and were all non-Hispanic white. Our results may not be representative of the broader population of competitive swimmers in the US, and further studies are needed to address these sampling limitations.

In conclusion, this first study of nutritional intake among adolescent swimmers in the US identifies several potential nutritional deficiencies that may jeopardize athletic performance and promote future disease risk, including osteoporosis. These deficiencies include higher than recommended fat intake and inadequate amounts of calcium, vitamin D, and daily servings of fruits, vegetables, grains, and dairy products. These results should be followed up in this population, on more recent samples, and utilizing more rigorous dietary assessment tools.

## Figures and Tables

**Table 1:** Characteristics of swimmers and non-swimmers.

Variable	Swimmers(1)	Non-athletes(2)	p*
	%	%	

**Gender (% male)**	32.9	39.0	0.386
**Pubertal Status**			
**% Early**	44.0	26.0	0.009
**% Late**	56.0	74.0	

	Mean ± s	Mean ± s	

**Age (years)**	13.7 *±* 2.5	14.4 *±* 2.8	0.058
**Weight (kg)**	52.8 *±* 14.4	59.8 *±* 18.5	0.003
**BMI (kg/m^2^)**	19.9 *±* 3.5	22.1 *±* 4.9	<0.0001
**Energy Expenditure per Day (kcal/day)**	2398.3 *±* 784.0	2195.7 *±* 723.4	0.066
**Energy Expenditure per kg per Day (kcal/kg/day)**	44.7 *±* 7.5	36.8 *±* 5.7	<0.0001

* *p*-values from between group t-test or chi-square;

(1) sample size varies from 98 to 100 and

(2) sample size varies from 89 to 91 due to missing data.

**Table 2:** Nutrient intake according to swim status and gender.

	Males			Females		

	Swimmers	Non-swimmers		Swimmers	Non-Swimmers	

	Mean ± s	Mean ± s	*p*	Mean ± s	Mean ± s	*p*

**Total Energy Intake (kcal/day)**	2570 ± 779	2421 ± 1024	0.527	2121 ± 881	1885 ± 795	0.141
**Total Energy Intake (kcal/kg body mass/day)**	50 ± 19	40.0 ± 19	0.056	44 ± 20	37 ± 19	0.082

	**Macronutrients (% of Total Energy)**					

Fat	36 ± 4	36 ± 6	0.931	36 ± 7	36 ± 7	0.879
Saturated Fat	12 ± 2	12 ± 2	0.839	11 ± 3	12 ± 3	0.826
Carbohydrate	52 ± 6	52 ± 8	0.785	52 ± 8	52 ± 8	0.976
Protein	14 ± 3	13 ± 2	0.219	14 ± 3	14 ± 3	0.209

	**Macronutrients (g/kg body mass)**					

Fat	2.0 ± 0.8	1.6 ± 0.9	0.122	1.8 ± 1.0	1.5 ± 0.9	0.127
Saturated Fat	0.6 ± 0.3	0.5 ± 0.3	0.105	0.6 ± 0.3	0.5 ± 0.3	0.159
Carbohydrate	6.4 ± 2.6	5.1 ± 2.4	0.049	5.5 ± 2.3	4.8 ± 2.6	0.118
Protein	6.4 ± 2.6	1.7 ± 0.8	1.3 ± 0.7	0.041	1.5 ± 0.7	0.027

	**Micronutrients (mg)**					

Iron	20 ± 7	16 ± 8	0.033	15 ± 7	14 ±7	0.156
Zinc	13 ± 5	11 ± 6	0.212	11 ± 5	9 ± 4	0.088
Vitamin D (μg)	7 ± 4	6 ± 4	0.289	5 ± 4	4 ± 3	0.191
Calcium (FFQ)	1197 ± 469	1086 ± 542	0.400	1117 ± 521	852 ± 397	0.003
Calcium (RAM)	1132 ± 456	1148 ± 591	0.904	1160 ± 625	737 ± 388	<0.001
Fiber (g)	17 ± 1	15 ±1	0.232	17 ± 1	15 ± 1	0.216
Cholesterol (mg)	221 ± 12	201 ± 12	0.226	224 ± 12	198 ± 12	0.125

	**Servings per Day**					

Vegetables	2.0 ± 1.3	2.1 ± 2.0	0.677	2.3 ± 1.7	2.2 ± 1.5	0.680
Grains	7.4 ± 2.8	6.1 ± 2.8	0.060	6.1 ± 3.4	5.4 ± 3.8	0.297
Fruits	1.3 ± 0.6	1.2 ± 0.8	0.356	1.7 ± 1.1	1.3 ± 1.0	0.030
Dairy	2.6 ± 1.5	2.3 ±1.5	0.437	2.4 ± 1.6	1.7 ± 1.1	0.012

Note: Due to missing data, sample size of mean nutrients range from 31 - 37 for male non-swimmers, 55 to 59 for female swimmers, and 54 to 61 for female non-swimmers. Male swimmers (n = 29) do not have any missing data.

**Table 3:** Adequacy of micronutrient intake, according to the dietary reference intake.

Nutrients	Adequacy (%)	Males		Females	

		Swimmers N = 30	Non-athletes N = 39	Swimmers N = 61	Non-Athletes N = 61
**Iron**	Inadequate	7	28	18	23
	Uncertain	0	10	20	26
	Adequate	93	62	62	51
**Zinc**	Inadequate	16	46	30	44
	Uncertain	7	21	10	13
	Adequate	77	33	60	43
**Calcium**	Inadequate	50	72	57	82
	Uncertain	20	3	15	8
	Adequate	30	25	28	10
**Vitamin D**	Inadequate	83	90	87	96
	Uncertain	10	10	10	2
	Adequate	7	0	3	2

## References

[1] USA Swimming (2010). General membership information.

[2] Berning JR, Troup JP, VanHandel PJ, Daniels J, Daniels N (1991). The nutritional habits of young adolescent swimmers. International Journal of Sports Nutrition.

[3] Hawley JA, Williams MM (1991). Dietary intakes of age-group swimmers. British Journal of Sports Medicine.

[4] Rodriguez NR, DiMarco NM, Langley S (2009). Position of the American Dietetic Association, Dietitians of Canada, and the American College of Sports Medicine: Nutrition and athletic performance. Journal of the American Dietetic Association.

[5] Story M, Stang J, Stang J, Story M (2005). Nutrition needs of adolescents. Guidelines for Adolescent Nutrition Services.

[6] Farajian P, Kavouras SA, Yannakoulia M, Sidossis LS (2004). Dietary intake and nutritional practices of elite Greek aquatic athletes. International Journal of Sport Nutrition and Exercise Metabolism.

[7] Kabasakalis A, Kalitsis G, Tsalis G, Mougios V (2007). Imbalanced nutrition of top-level swimmers. International Journal of Sports Medicine.

[8] Paschoal VCP, Amancio OMS (2004). Nutritional status of Brazilian elite swimmers. International Journal of Sports Nutrition and Exercise Metabolism.

[9] Meyer F, O’Connor H, Shirreffs SM (2007). Nutrition for young athlete. Journal of Sports Sciences.

[10] Hassapidou MN, Valasiadou V, Tzioumakis L, Vrantza P (2002). Nutrient intake and anthropometric characteristics of adolescent Greek swimmers. Nutrition and Dietetics.

[11] Martinez S, Pasquarelli BN, Romaquera D, Arasa C, Tauler P, Aquilo A (2011). Anthropometric characteristics and nutritional profile of young amateur swimmers. Journal of Strength and Conditioning Research.

[12] Marshall WA, Tanner JM (1969). Variations in patterns of pubertal changes in girls. Archives of Disease in Childhood.

[13] Marshall WA, Tanner JM (1969). Variations in patterns of pubertal changes in boys. Archives of Disease in Childhood.

[14] Duke PM, Litt IF, Gross RT (1980). Adolescents’ self-assessment of sexual maturation. Pediatrics.

[15] He Q, Horlick M, Fedun B, Wang J, Pierson RN, Heska S, Gallagher D (2002). Trunk fat and blood pressure in children through puberty. Circulation.

[16] Sallis JF, Haskell WL, Wood PD, Fortmann SP, Rogers T, Blair SN, Paffenbarger RS (1985). Physical activity assessment methodology in the Five-City Project. American Journal of Epidemiology.

[17] Sallis JF, Buono MJ, Roby JL (1993). Seven-day recall and other physical activity self-reports in children and adolescents. Medicine and Science in Sports and Exercise.

[18] Block G, Hartman AM, Dresser CM, Carroll MD, Gannon J, Gardner L (1986). A data-based approach to diet questionnaire design and testing. American Journal of Epidemiology.

[19] Boucher B, Cotterchio M, Kreiger N, Nadalin V, Block T, Block G (2006). Validity and reliability of the Block98 food-frequency questionnaire in a sample of Canadian women. Public Health Nutrition.

[20] Ochner CN, Lowe MR (2007). Self-reported changes in dietary calcium and energy intake predict weight regain following a weight loss diet in obese women. The Journal of Nutrition.

[21] Wilson AM, Lewis RD (2004). Disagreement of energy and macronutrient intakes estimated from a food frequency questionnaire and 3-day diet record in girls 4 to 9 years of age. Journal of the American Dietetic Association.

[22] Hertzler AA, Frary RB (1994). A dietary calcium rapid assessment method (RAM). Topics in Clinical Nutrition.

[23] Ward KD, Mays KE, Berg MB, Slawson DA, Vukadinovich CM, McClanahan BS, Clemens LH (2004). Reliability and validity of a brief questionnaire to assess calcium intake in female collegiate athletes. International Journal of Sport Nutrition and Exercise Metabolism.

[24] Institute of Medicine (2006). Dietary reference intake: The essential guide to nutrient requirements.

[25] Institute of Medicine (2011). Dietary Reference Intakes for calcium and vitamin D.

[26] US Department of Agriculture (2011). Economic research service nutrient availability data.

[27] Jeukendrup A, Cronin L (2011). Nutrition and elite young athletes. Medicine and Sport Science.

[28] Burke LM, Cox GR, Culmmings NK, Desbrow B (2001). Guidelines for daily carbohydrate intake: Do athletes achieve them?. Sports Medicine.

[29] Roger VL, Go AS, Lloyd-Jones DM, Benjamin EJ, Berry JD, Borden WB, Turner MB (2012). Heart disease and stroke statistics—2012 update: A report from the American Heart Association. Circulation.

[30] Bailey DA, McKay HA, Mirwald RL, Crocker PR, Faulkner RA (1999). A six-year longitudinal study of the relationship of physical activity to bone mineral accrual in growing children: The University of Saskatchewan bone mineral accrual study. Journal of Bone and Mineral Research.

[31] Morel J, Combe B, Francisco J, Bernard J (2001). Bone mineral density of 704 amateur sportsmen involved in different physical activities. Osteoporosis International.

[32] Nichols DL, Sanborn CF, Bonnick SL, Gench B, DiMarco N (1995). Relationship of regional body composition to bone mineral density in college females. Medicine and Science in Sports and Exercise.

[33] Dias Quiterio AL, Carnero EA, Baptista FM, Sardinha LB (2011). Skeletal mass in adolescent male athletes and nonathletes: Relationships with high-impact sports. Journal of Strength and Conditioning Research.

[34] Huncharek M, Muscat J, Kupelnick B (2008). Impact of dairy products and dietary calcium on bone-mineral content in children: Results of a meta-analysis. Bone.

[35] Tylavsky FA, Ryder KA, Lyytikainen A, Cheng S (2005). Vitamin D parathyroid hormone, and bone mass in adolescents. The Journal of Nutrition.

[36] McDonald R, Keen CL (1988). Iron, zinc and magnesium nutrition and athletic performance. Sports Medicine.

[37] Yamaguchi M (1998). Role of zinc in bone formation and bone resorption. Journal of Trace Elements in Experimental Medicine.

[38] Medeiros DM, Plattner A, Jennings D, Stoecker B (2002). Bone morphology, strength and density are compromised in iron-deficient rats and exacerbated by calcium restriction. The Journal of Nutrition.

[39] Center for Disease Control and Prevention (2011). National health and nutrition examination survey.

[40] Burrows TL, Martin RJ, Collins CE (2010). A systematic review of the validity of dietary assessment methods in children when compared with the method of doubly labeled water. Journal of the American Dietetic Association.

